# LncRNA MEG8 promotes NSCLC progression by modulating the miR-15a-5p-miR-15b-5p/PSAT1 axis

**DOI:** 10.1186/s12935-021-01772-8

**Published:** 2021-02-01

**Authors:** Kai Guo, Di Qi, Bo Huang

**Affiliations:** Department of Thoracic Surgery, The First Affiliated Hospital of Jinzhou Medical University, Renming Street #5-2, Guta District, Jinzhou City, Liaoning Province 121000 People’s Republic of China

**Keywords:** NSCLC, Progression, lncRNA MEG8, miR-15a-5p, miR-15b-5p, PSAT1

## Abstract

**Background:**

Non-small cell lung cancer (NSCLC) is the most common tumor with severe morbidity and high mortality. Long non-coding RNAs (lncRNAs) as crucial regulators participate in multiple cancer progressions. However, the role of lncRNA MEG8 in the development of NSCLC remains unclear. Here, we aimed to investigate the effect of lncRNA MEG8 on the progression of NSCLC and the underlying mechanism.

**Methods:**

Cell proliferation was analyzed by EdU assays. The impacts of lncRNA MEG8, miR-15a-5p, and miR-15b-5p on cell invasion and migration of NSCLC were assessed by transwell assay. The luciferase reporter gene assay was performed using the Dual-luciferase Reporter Assay System. The effect of lncRNA MEG8, miR-15a-5p, and miR-15b-5p on tumor growth was evaluated in nude mice of Balb/c in vivo.

**Results:**

We revealed that the expression levels of MEG8 were elevated in the NSCLC patient tissues compared to that in adjacent normal tissues. The expression of MEG8 was negatively relative to that of miR-15a-5p and miR-15b-5p in the NSCLC patient tissues. The expression of MEG8 was upregulated, while miR-15a-5p and miR-15b-5p were downregulated in NSCLC cell lines. The depletion of MEG8 inhibited NSCLC cell proliferation, migration, and invasion in vitro*.* MEG8 contributed to NSCLC progression by targeting miR-15a-5p/miR-15b-5p in vitro*.* LncRNA MEG8 contributes to tumor growth of NSCLC via the miR-15a/b-5p/PSAT1 axis in vivo*.* Thus, we concluded that lncRNA MEG8 promotes NSCLC progression by modulating the miR-15a/b-5p/PSAT1 axis.

**Conclusions:**

Our findings demonstrated that lncRNA MEG8 plays a critical role in NSCLC development. LncRNA MEG8, miR-15a-5p, miR-15b-5p, and PSAT1 may serve as potential targets for NSCLC therapy.

## Background

Lung cancer is the most prevalent malignancy and is the leading cause of tumor-associated mortality globally, according to the latest annual statistics report of global cancer [1]. Non-small cell lung cancer (NSCLC) accounts for about 83% of primary lung cancer [2]. Even with advancements in surgical and chemotherapeutic interventions, the 5-year survival for NSCLC cases remains poor, and the recurrence incidence is high because of chemoresistance or tumor metastasis [3]. Understanding of the mechanism underlying the NSCLC progression will benefit the diagnosis, therapy, and prognosis for NSCLC [4].

Long non-coding RNAs (lncRNAs) as the emerging critical biological regulators, play critical roles in many physiological and pathological processes such as cell cycle, differentiation, apoptosis, cardiovascular diseases, and cancer progression [5, 6]. Many lncRNAs are identified to participate in the modulation of NSCLC. LncRNA linc00673 mediates the epithelial–mesenchymal transition, proliferation, invasion, and migration of NSCLC by modulating miR-150-5p [7]. LncRNA MALAT1 promotes the progression of NSCLC by regulating the miR-200a-3p/programmed death-ligand-1 signaling [8]. LncRNA-HIT increases NSCLC cell proliferation by regulating the expression of E2F1 [9]. LncRNA MEG8 is a poor-investigated lncRNAs. It has been reported that lncRNA MEG8 enhances the epithelial-mesenchymal transition by modulating epigenetic progression in pancreatic and lung cancer cells [10, 11]. LncRNA MEG8 is abnormally expressed in lung cancer tissues [12]. LncRNA MEG8 suppresses activation of hepatic stellate cells and epithelial-mesenchymal transition of hepatocytes via the Notch pathway [10, 11]. However, the effect of lncRNA MEG8 on NSCLC progression and the underlying mechanism are unclear.

MicroRNAs (miRNAs) are short (20–25 nucleotides) non-coding RNAs that exert significant functions in numerous biological processes [13]. MiRNAs regulate gene expression at post-transcriptional levels by pairing with target mRNAs at the 3′ untranslated region (3′-UTR) [14]. MiRNAs modulate different targets that hold essential functions in a broad spectrum of biological and medical processes, including cell apoptosis, proliferation, differentiation, invasion, metastasis, and tumorigenesis [15]. Studies have revealed that miRNAs are involved in the progression of NSCLC. MicroRNA-340-5p represses the growth and metastasis of NSCLC cells by modulating ZNF503 [16]. MiR-1269a serves as an onco-miRNA in NSCLC by inhibiting the expression of SOX6 [17]. MiR-744-5p restrains the proliferation and invasion of NSCLC through targeting PAX2 [18]. Meanwhile, previous investigations identified that miR-15a/b-5p played crucial role in modulating tumorigenesis by interacting with lncRNAs and targeted genes. MiR-16-5p, miR-15a-5p and miR-15b-5p repress neuroblastoma progression through directly regulation of MYCN [19]. LncRNA-H19 stimulates the CDC42/PAK1 signaling to increase cell proliferation, invasion, and migration of hepatocellular carcinoma by sponging miR-15b [20]. LncRNA TTN-AS1 serves as a sponge of miR-15b-5p to regulate the expression of FBXW7 in ovarian cancer [21]. MiR-15a-5p is identified as a new prognosis marker, foretelling the recurrence of colorectal adenocarcinoma [22]. MiR-15a-5p overcomes the cell growth of endometrial cancer through the Wnt/β-catenin pathway by repressing WNT3A [23]. Furthermore, it has been found that miR-15a-5p and miR-15b-5p are down-regulated in NSCLC patients and can serve as potential diagnostic biomarkers for NSCLC [24, 25]. However, the specific role of miR-15a/b-5p in the development of NSCLC is unknown.

Phosphoserine aminotransferase 1 (PSAT1) modulates the progress of 3-phosphohydroxy-pyruvate converging to 3-phosphoserine in l-serine biosynthesis [26]. Serine presents a vital function in regulating the development of the central nervous system [27]. Patients with PSAT1 deficiency exhibit rigid neurological abnormalities [28]. The deficient serine synthesis due to PSAT1 loss is further correlated with Neu-Laxova syndrome (NLS), an uncommon autosomal-recessive disease defined by a recognizable model of severe abnormalities driving to early postnatal or prenatal lethality [29, 30]. PSAT1 also contributes to the modulation of cancer progression. ATF4-mediated PSAT1 increases cell proliferation of ER-negative breast cancer through the GSK3β/cyclin D1 pathway [31]. Elevated levels of PSAT1 in the tumor tissues is correlated with the prognosis of ovarian cancer [32]. MicroRNA-195-5p reduces angiogenesis and cisplatin resistance of ovarian cancer by restraining the PSAT1-mediated GSK3β/β-catenin pathway [33]. Moreover, a recent study showed that enhancement of PSAT1 increased NSCLC metastasis through repressing the IRF1-IFNγ signaling [34]. PSAT1 regulates the degradation of cyclin D1 and promotes NSCLC cell proliferation [35]. However, the relationship among PSAT1, lncRNA MEG8 and miR-15a/b-5p in the NSCLC progression remains unclear.

In this study, we aimed to explore the role of lncRNA MEG8 in the development of NSCLC. We identified a novel function of lncRNA MEG8 in promoting NSCLC progression by regulating the miR-15a/b-5p/PSAT1 axis.

## Methods

### NSCLC clinical samples

A total of 37 NSCLC clinical samples were obtained from the First Affiliated Hospital of Jinzhou Medical University between June 2016 and Mar 2018. All the patients were diagnosed by histopathological analysis. All cases were independently diagnosed and reviewed by two clinicians. Before surgery, no systemic or local therapy was carried out in the subjects. The NSCLC tissues and corresponding para-neoplastic tissues obtained from the patients were immediately frozen with liquid nitrogen, followed by storing at − 80 °C before use. The written consent form was obtained from all patients and healthy cases. This study conformed to the experimental guidelines of the World Medical Association and the Ethics Committee of the First Affiliated Hospital of Jinzhou Medical University.

### Cell culture and treatment

The 16HBE, A549, H1299, H1975, SPC-A1, and PC-9 cell lines were purchased from American Type Tissue Culture Collection in 2019. Cells were authenticated by STR analysis recently. Cells were cultured in DMEM (Solarbio, China) (16HBE, A549, H1299, PC-9) or RPMI-1640 (Solarbio, China) (H1975, SPC-A1) containing 10% fetal bovine serum (Gibco, USA), 0.1 mg/mL streptomycin (Solarbio, China) and 100 units/mL penicillin (Solarbio, China) at 37 °C with 5% CO_2_.

### Cell transfection

The MEG8 shRNA and control shRNA, PSAT1 siRNA and control siRNA, miR-15a-5p mimic, miR-15b-5p mimic, control mimic, miR-15a-5p inhibitor, miR-15b-5p inhibitor, and control inhibitor were used in this study (GenePharma, China). Cell transfection was performed by Liposome 3000 (Invitrogen, USA) following the manufacturer's instructions. The MEG8 shRNA targeted sequence was: GGAAUAGACGAGAUUGGAU; The PSAT1 siRNA targeted sequence was: ACTCAGTGTTGTTAGAGAT; the sequence of miR-15a-5p was: 5′-UAGCAGCACAUAAUGGUUUGUG-3′; the sequence of miR-15b-5p was: 5′-UAGCAGCACAUCAUGGUUUACA-3′.

### Quantitative reverse transcription-PCR (qRT-PCR)

Total RNAs were extracted by TRIZOL (Invitrogen, USA) and reverse transcribed into cDNA following the manufacturer's instructions (Thermo, USA). The qRT-PCR was carried out using SYBR Real-time PCR I kit (Takara, Japan). The standard control for miRNA and mRNA/lncRNA was U6 and GAPDH, respectively. Quantitative determination of the RNA levels was conducted in triplicate independent experiments. The primer sequences are as follows:

LncRNA MEG8 forward: 5′-CTTGCTTCCTGGCACGAG-3′.

LncRNA MEG8 reverse: 5′-CAGGAAACAGCTATGAC-3′.

miR-15a-5p forward: 5′-TAGCAGCACATAATGGTTTGTG-3′.

miR-15a-5p reverse: 5′-CACAAACCATTATGTGTCTGCTA-3′.

miR-15b-5p forward: 5′-ATGAACTTTCTCTGTCTTGG-3′.

miR-15b-5p reverse: 5′-CAGTGCGTGTCGTGGAGT-3′.

PSAT1 forward: 5′-GTCCAGTGGAGCCCCAAAA-3′.

PSAT1 reverse: 5′-TGCCTCCCACAGACCTATGC-3′.

GAPDH forward: 5′-AAGAAGGTGGTGAAGCAGGC-3′.

GAPDH reverse: 5′-TCCACCACCCAGTTGCTGTA-3′.

U6 forward: 5′-GCTTCGGCAGCACATATACTAA-3′.

U6 reverse: 5′-AACGCTTCACGAATTTGCGT-3′.

### CCK-8 assays

Cell viability was assessed by CCK-8 assays. About 5 × 10^3^ cells were put into 96 wells and cultured for 12 h. Cells were then used for transfection or treatment. After 0 h, 24 h, 48 h, 72 h, and 96 h, CCK-8 solution (KeyGEN Biotech, China) was added and cells were cultured at 37 °C for another 2 h. The ELISA browser was applied to analyze the absorbance at 450 nm (Bio-Tek EL 800, USA).

### Edu assays

Cell proliferation was analyzed by EdU assays using EdU detecting kit (RiboBio, China). Briefly, NSCLC cells were cultured with EdU for 2 h, followed by paraformaldehyde fix (4%, room temperature, 30 min). Cells were then permeabilized with Triton X-100 (0.4%, 10 min) and stained with staining cocktail of EdU in the dark (room temperature, 30 min). Next, nuclear of the cells were stained with Hoechst (room temperature, 30 min), and images were analyzed using a fluorescence microscope.

### Transwell assays

Transwell assays analyzed the effect of lncRNA MEG8, miR-15a-5p, and miR-15b-5p on cell invasion and migration of NSCLC using a Transwell plate (Corning, USA) following the manufacturer’s instructions. Briefly, the upper chambers were plated with around 1 × 10^5^ cells, solidified through 4% paraformaldehyde, and stained with crystal violet. The invaded and migrated cells were recorded and calculated.

### Analysis of cell apoptosis

About 2 × 10^5^ cells were plated on 6-well dishes. Cell apoptosis was analyzed using the Annexin V-FITC Apoptosis Detection Kit (CST, USA) following the manufacturer’s instructions. Briefly, about 2 × 10^6^ collected and washed cells collected by binding buffer and were dyed at 25 ℃, followed by flow cytometry analysis.

### Luciferase reporter gene assay

Luciferase reporter gene assays were performed using the Dual-luciferase Reporter Assay System (Promega, USA). Briefly, cells were treated with miR-15a-5p mimic, miR-15b-5p mimic or control mimic, the vector containing MEG8, MEG8 mutant, PSAT1, and PSAT1 mutant fragment were transfected into the cells using Lipofectamine 3000 (Invitrogen, USA), followed by analysis of luciferase activities, in which Renilla was applied as a normalized control.

### RNA pull-down

Biotin-marked RNAs were transcribed using biotin-UTP of MEGAscript T7 Kit (Thermo, USA) in vitro and purified by MEGAclear Kit (Thermo, USA), followed by incubation with entire cell lysates. Biotin-labeled transcripts and interacted RNAs were isolated with streptavidin beads and then subjected to qPCR analysis.

### Western blot analysis

Total proteins were extracted from the cells or mice tissues with RIPA buffer (CST, USA). Protein concentrations were measured using the BCA Protein Quantification Kit (Abbkine, USA). Same amount of protein samples were separated by SDS-PAGE (12% polyacrylamide gels), followed by transferring to PVDF membranes (Millipore, USA). The membranes were hindered with 5% milk and hatched at 4 °C overnight with the primary antibodies for PSAT1 (1:1000) (Proteintech, China) and GAPDH (1:1000) (Beyotime Biotechnology, China. Then, the corresponding second antibodies (1:1000) (Beyotime Biotechnology, China) were used for hatching the membranes at room temperature for 1 h, followed by visualization using an Odyssey CLx Infrared Imaging System.

### Analysis of tumorigenicity in nude mice

The effect of lncRNA MEG8, miR-15a-5p, and miR-15b-5p on tumor growth was analyzed in nude mice of Balb/c in vivo. We randomly separated the mice into four groups (n = 5). To establishment the in vivo tumor model, A549 cells were treated with control shRNA (Ctrl), MEG8 shRNA (shMEG8), or co-treated with MEG8 shRNA (shMEG8) and the inhibitor of miR-15b-5p and miR-15a-5p, respectively. And about 1 × 10^7^ cells were subcutaneously injected into the mice. After 7 days of injection, tumor growth was measured every 7 days. The mice were sacrificed after 28 days of injection, and tumors were scaled. Tumor volume (V) was observed by estimating the length and width with calipers and measured with the method × 0.5. The expression levels of Ki-67 of the tumor tissues were determined by immunohistochemical staining with the Ki67 antibody (Santa Cruz Biotechnology, USA). The protein expression levels of PSAT1 (1:1000) (Proteintech, China) and GAPDH (1:1000) (Beyotime Biotechnology, China) were analyzed by Western blot analysis in the tumor tissues. The expression of miR-15-5p was measured by qPCR in the tumor tissues. Animal care and method procedure were authorized by the Animal Ethics Committee.

### Statistical analyses

Data were presented as mean ± SD, and statistical analysis was performed by GraphPad prism 7. The unpaired Student’s *t* test was applied for comparing two groups, and one-way ANOVA was applied for comparing multiple groups. *P* < 0.05 was considered as statistically significant.

## Results

### MEG8 is up-regulated and miR-15a-5p/miR-15b-5p is down-regulated in NSCLC patients and NSCLC cell lines

**Fig. 1:**
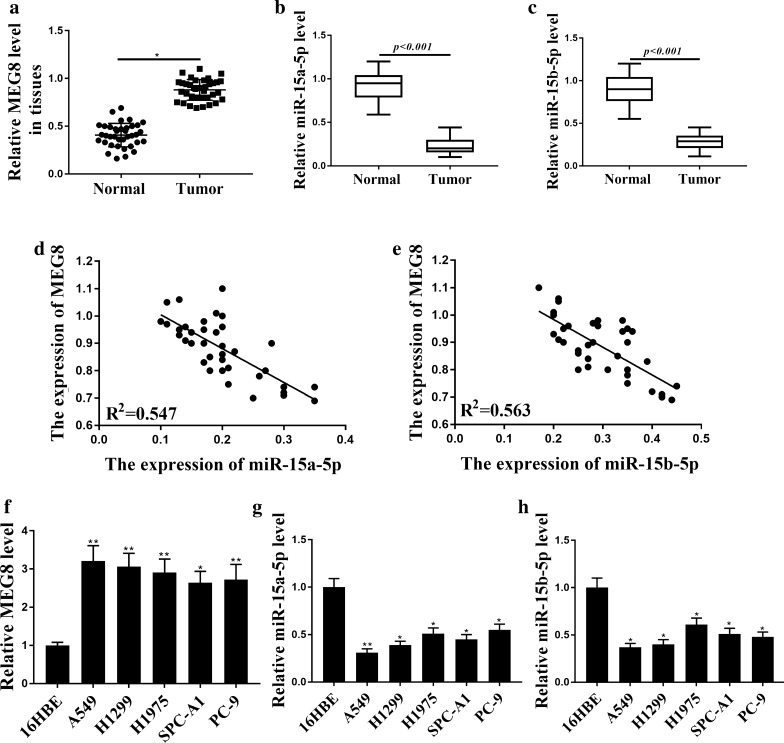
MEG8 is up-regulated and miR-15a-5p/miR-15b-5p is down-regulated in the NSCLC patients and NSCLC cell lines. **a** The expression levels of lncRNA MEG8 were measured by qPCR in the NSCLC patient tissues (n = 37) and the adjacent normal tissues (n = 37). **b**, **c** The expression of miR-15a-5p (**b**) and miR-15b-5p (**c**) was tested by qPCR in the NSCLC patient tissues (n = 37) and the adjacent normal tissues (n = 37). **d**, **e** The correlation of MEG8 with miR-15a-5p (**d**) and miR-15b-5p (**e**) was analyzed by qPCR in the NSCLC patient tissues (n = 37). **f**–**h** The expression levels of MEG8 (F), miR-15a-5p (**g**), and miR-15b-5p (**h**) were assessed by qPCR in the 16HBE, A549, H1299, H1975, SPC-A1, and PC-9 cells. Data are presented as mean ± SD. Statistic significant differences were indicated: *P < 0.05, **P < 0.01

To assess the potential correlation of lncRNA MEG8 and miR-15a/b-5p with NSCLC progression, their expression was evaluated in NSCLC patients and NSCLC cell lines. It showed that the expression levels of MEG8 were significantly elevated in NSCLC patient tissues (n = 37) compared to that in the adjacent normal tissues (n = 37) (*P* < 0.05) (Fig. 1a), implying that MEG8 is associated with the clinical development of NSCLC. The expression of miR-15a-5p (*P* < 0.01) (Fig. 1b) and miR-15b-5p (*P* < 0.01) (Fig. 1c) was down-regulated in NSCLC patient tissues (n = 37) compared to that in the adjacent normal tissues (n = 37). Meanwhile, the expression of MEG8 was negatively corelated with miR-15a-5p (R^2^ = 0.547) (Fig. 1d) and miR-15b-5p (R^2^ = 0.563) (Fig. 1e) in NSCLC patient tissues (n = 37), indicating the potential interplay of MEG8 and miR-15a/b-5p in the NSCLC progression. Besides, the expression of MEG8 was also upregulate (*P* < 0.01) (Fig. 1f), while the expression of miR-15a-5p (*P* < 0.01) (Fig. 1g) and miR-15b-5p (*P* < 0.05) (Fig. 1h) was downregulated in the NSCLC cell lines, including A549, H1299, H1975, SPC-A1 and PC-9, compared to that in the human normal pneumonocyte 16HBE cells, further confirming the potential correlation of MEG8 and miR-15a/b-5p with the NSCLC development.

### The depletion of lncRNA MEG8 reduces NSCLC cell proliferation, migration and invasion in vitro

**Fig. 2:**
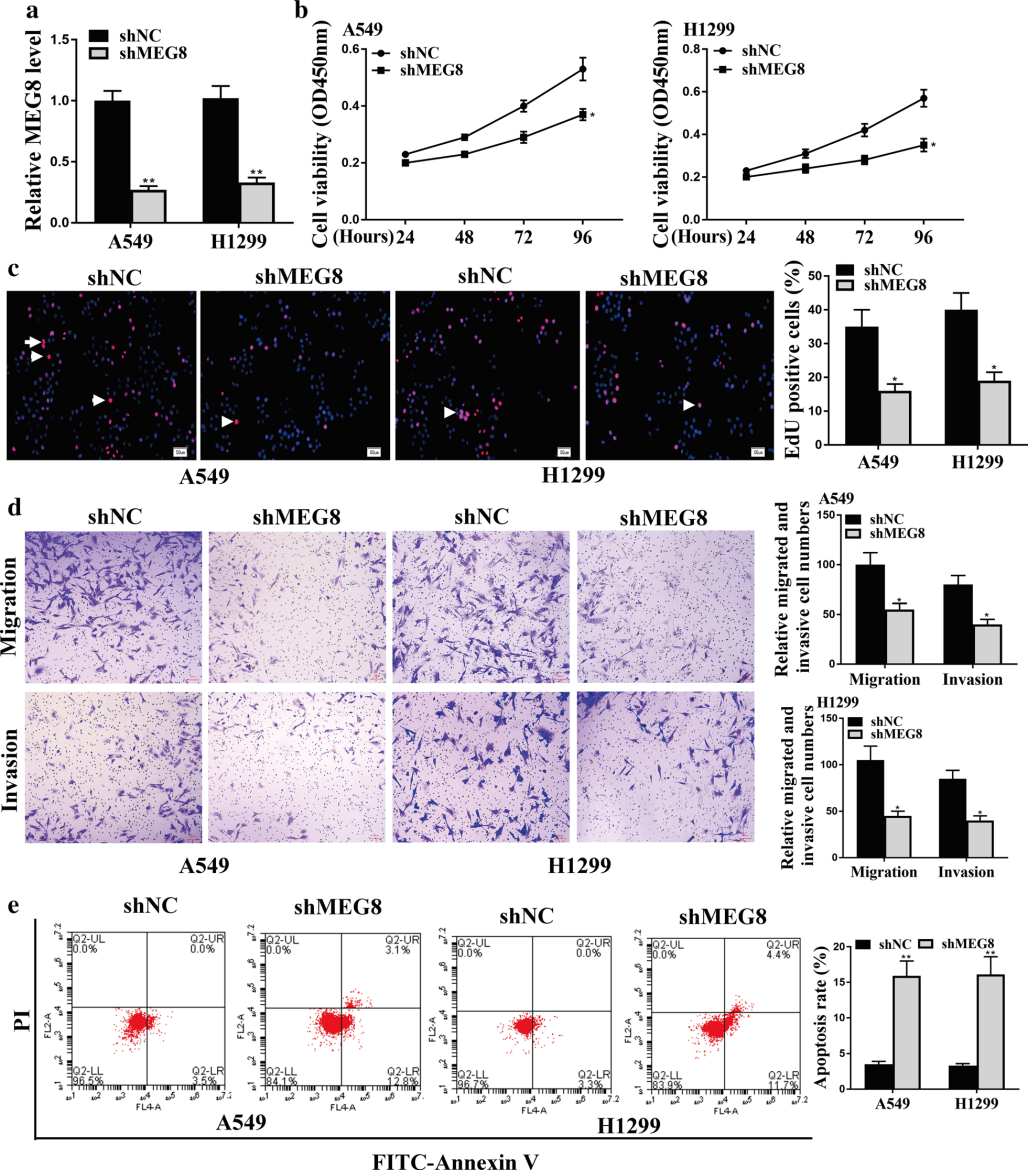
The depletion of lncRNA MEG8 reduces NSCLC cell proliferation, migration and invasion in vitro. **a**–**e** The A549 and H1299 cells were infected with the lentiviral plasmids carrying MEG8 shRNA (shMEG8) or corresponding control shRNA (shNC). **a** The expression levels of lncRNA MEG8 were tested by qPCR assays in the cells. **b** The cell viability was measured by CCK-8 assays in the cells. **c** The cell proliferation was tested by EdU assays in the cells, in which the pink represented EdU and the blue represented nuclear stained by Hoechst. **d** The cell migration and invasion were examined by transwell assays in the cells. **e** The cell apoptosis was measure by flow cytometry analysis in the cells, in which the x-axis represented the Annexin V-FITC and the y-axis represented the propidium iodide (PI). Data are presented as mean ± SD. Statistic significant differences were indicated: *P < 0.05, **P < 0.01

The effect of lncRNA MEG8 on the progression of NSCLC was further explored in vitro. The lentiviral plasmids carrying MEG8 shRNA (shMEG8) or corresponding control shRNA (shNC) were infected in the NSCLC A549 and H1299 cell lines. The knockdown efficiency of MEG8 shRNA was validated by qPCR assays (*P* < 0.01) (Fig. 2a). CCK-8 assays revealed that the depletion of MEG8 remarkably reduced the cell viability in A549 and H1299 cells (*P* < 0.05) (Fig. 2b). The EdU-positive cells were inhibited by the knockdown of MEG8 as well (*P* < 0.05) (Fig. 2c), suggesting that MEG8 is required for NSCLC cell proliferation. Moreover, transwell assays demonstrated that depletion of MEG8 impaired the migration and invasion of A549 and H1299 cells (*P* < 0.05) (Fig. 2d), indicating that MEG8 contributes to the NSCLC progression in vitro. Consistently, the depletion of MEG8 enhanced the expression of EMT markers, such as E-cadherin, N-cadherin, and Vimentin, in the A549 and H1299 cells (Additional file 1 Fig. 1A). Besides, the knockdown of MEG8 notably increased the apoptosis of A549 and H1299 cells (*P* < 0.01) (Fig. 2e), confirming the role of MEG8 in the development of NSCLC.

### LncRNA MEG8 interacts with miR-15a/b-5p in vitro

**Fig. 3:**
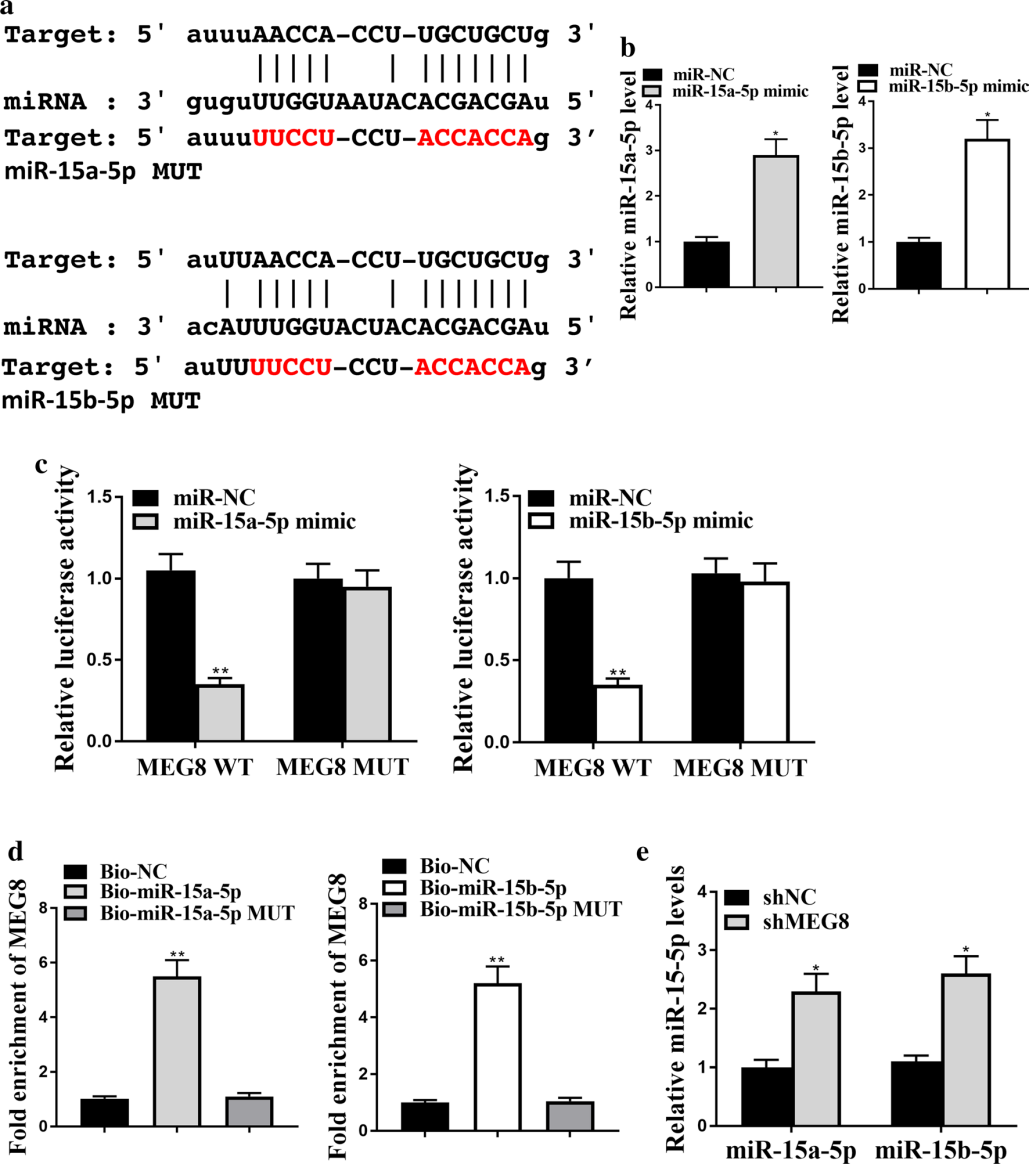
LncRNA MEG8 interacts with miR-15a/b-5p in vitro. **a** Potential interaction between lncRNA MEG8 and miR-15a/b-5p was identified by the bioinformatic analysis using ENCORI (http://starbase.sysu.edu.cn/index.php). **b** The expression levels of miR-15a-5p and miR-15b-5p were tested by qPCR in the A549 cells treated with control mimic (miR-NC), or miR-15a/b-5p mimics. **c** Luciferase activities of MEG8 (MEG8 WT) and MEG8 with the miRNA-binding site mutant (MEG8 MUT) were determined by luciferase reporter gene assays in the A549 cells treated with control mimic (miR-NC), miR-15a-5p mimic, or miR-15b-5p mimic, respectively. **d** The interaction of MEG8 with wild type miR-15a-5p (Bio-miR-15a-5p), miR-15a-5p with the MEG8-binding site mutant (Bio-miR-15a-5p MUT), wild type miR-15b-5p (Bio-miR-15b-5p), and miR-15b-5p with the MEG8-binding site mutant (Bio-miR-15b-5p MUT) was examined by RNA-pull down followed by qPCR in the A549 cells. **e** The expression levels of miR-15a-5p and miR-15b-5p were measured by qPCR in the A549 cells infected with the lentiviral plasmids carrying MEG8 shRNA (shMEG8) or corresponding control shRNA (shNC). Data are presented as mean ± SD. Statistic significant differences were indicated: *P < 0.05, **P < 0.01

Next, we identified the potential interaction between lncRNA MEG8 and miR-15a/b-5p with bioinformatic analysis using ENCORI (http://starbase.sysu.edu.cn/index.php) (Fig. 3a). A549 cells were treated with miR-15a-5p mimic or miR-15b-5p mimic, or the corresponding control mimic, and the efficiency was verified (*P* < 0.05) (Fig. 3b). The miR-15a-5p mimic along with miR-15b-5p mimic remarkably reduced the luciferase activities of MEG8 but not MEG8 with the miRNA-binding site mutant that is shown in Fig. 3a (*P* < 0.01) (Fig. 3c). RNA-pull down followed by qPCR further confirmed the interaction of MEG8 with miR-15a-5p and miR-15b-5p, but not the counterparts containing the MEG8-binding site mutant (*P* < 0.01) (Fig. 3d), indicating that lncRNA MEG8 interacts with miR-15a/b-5p. Moreover, depletion of MEG8 could enhance the expression of miR-15a-5p and miR-15b-5p in the cells (*P* < 0.05) (Fig. 3e), suggesting that MEG8 is able to target miR-15a/b-5p.

### LncRNA MEG8 promotes NSCLC progression by targeting miR-15a/b-5p in vitro

**Fig. 4:**
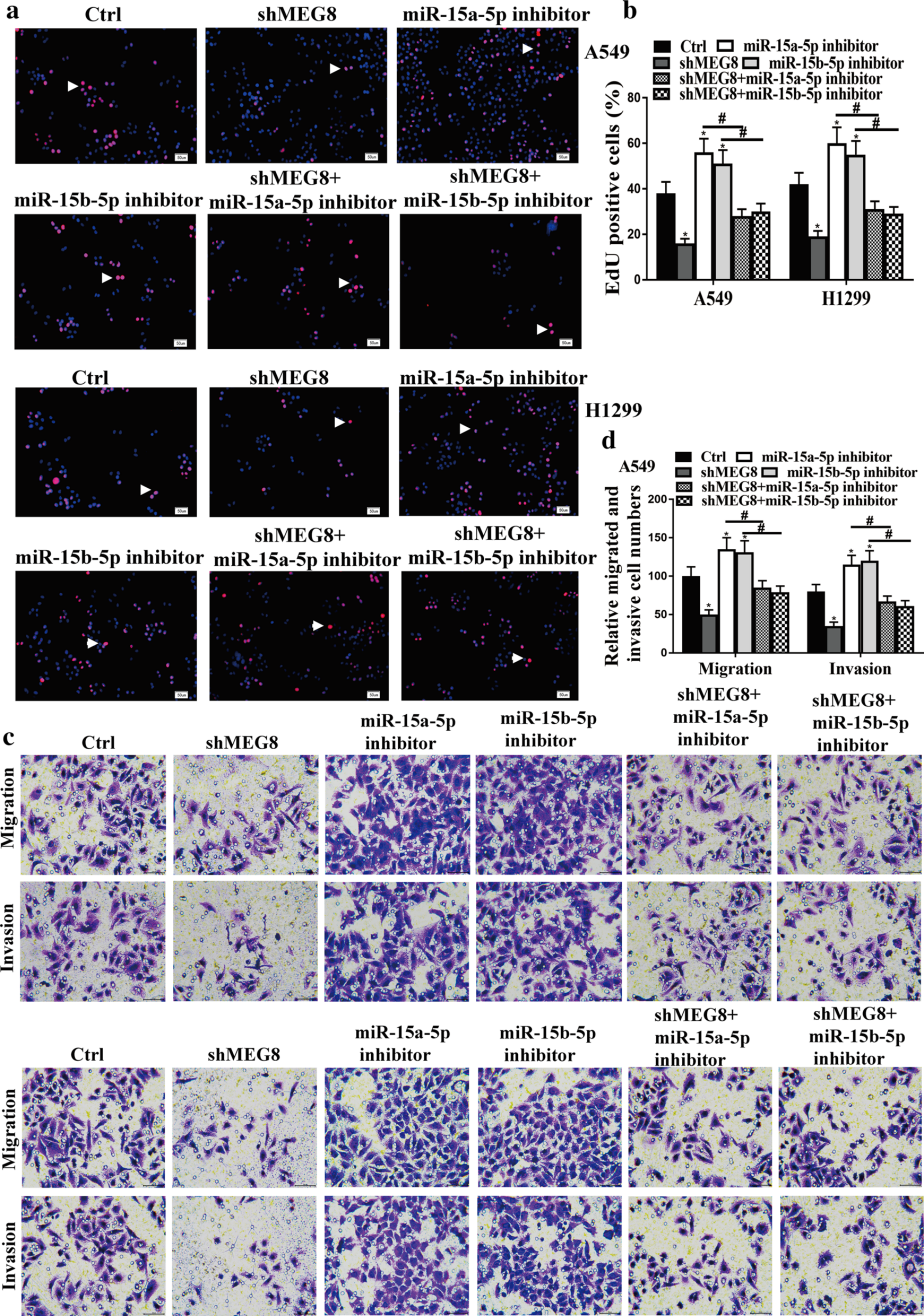
LncRNA MEG8 promotes NSCLC cell proliferation, migration, and invasion by targeting miR-15a/b-5p in vitro. **a**–**d** The A549 and H1299 cells were treated with control shRNA (Ctrl), MEG8 shRNA (shMEG8), miR-15a/b-5p inhibitors, or co-treated with MEG8 shRNA (shMEG8) and the inhibitors of miR-15a/b-5p, respectively. **b** The cell proliferation was tested by EdU assays in the cells. **c**, **d** The cell migration and invasion were examined by transwell assays in the cells. Data are presented as mean ± SD. Statistic significant differences were indicated: *P < 0.05, ^#^*P* < 0.05

**Fig. 5:**
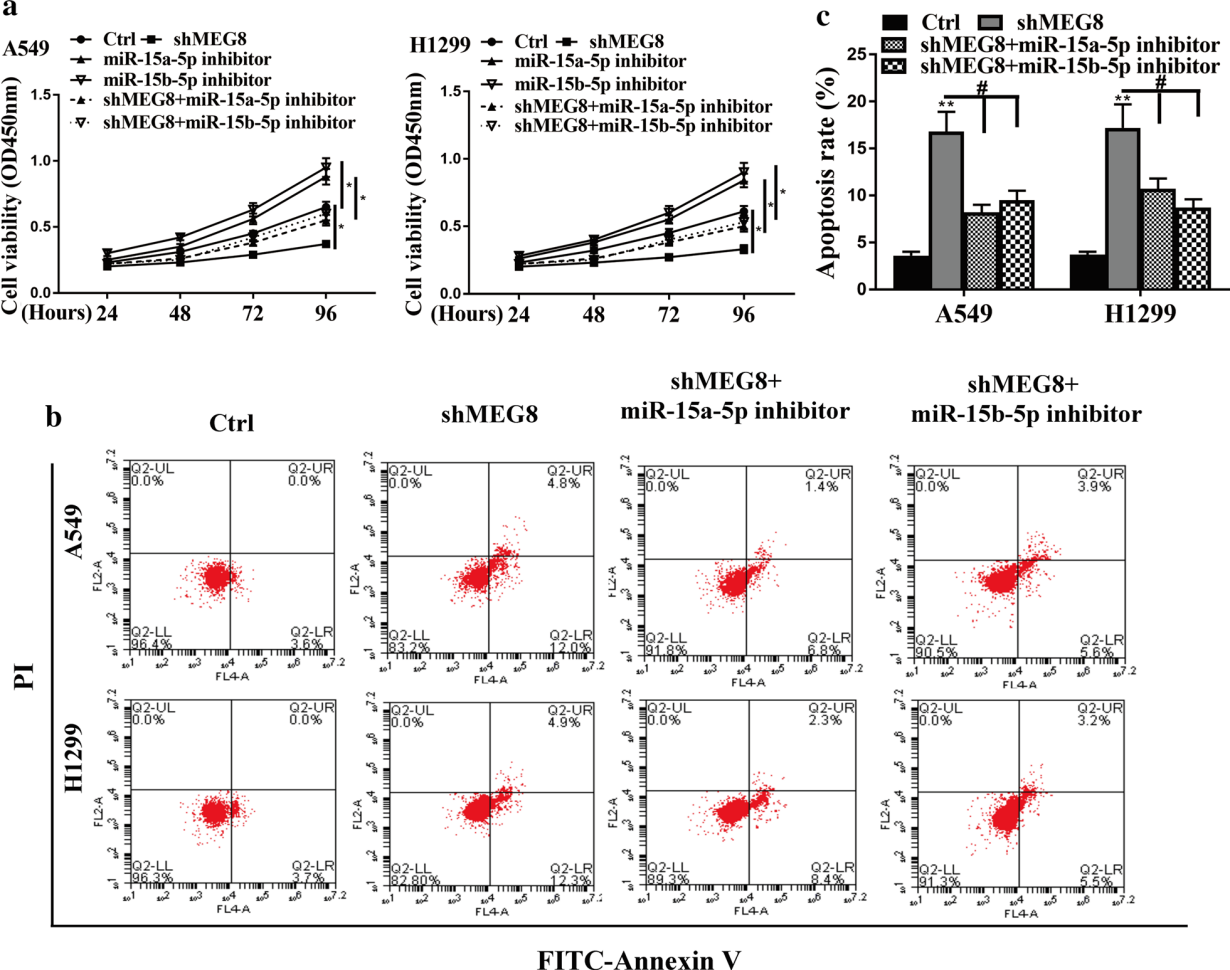
LncRNA MEG8 promotes NSCLC cell proliferation, migration, and invasion by targeting miR-15a/b-5p in vitro. **a**–**c** The A549 and H1299 cells were treated with control shRNA (Ctrl), MEG8 shRNA (shMEG8), miR-15a/b-5p inhibitors, or co-treated with MEG8 shRNA (shMEG8) and the inhibitors of miR-15a/b-5p, respectively. **a** Cell viability was measured by CCK-8 assays in the cells. **b**, **c** The cell apoptosis was measure by flow cytometry analysis in the cells, in which the x-axis represented the Annexin V-FITC and the y-axis represented the propidium iodide (PI). Data are presented as mean ± SD. Statistic significant differences were indicated: *P < 0.05, **P < 0.01, ^#^*P* < 0.05

The role of the lncRNA MEG8/miR-15a/b-5p axis in NSCLC development was then explored in vitro. EdU assays demonstrated that the depletion of MEG8 reduced, and inhibitors of miR-15a/b-5p enhanced the cell proliferation in A549 cells, in which inhibitor of miR-15a-5p and miR-15b-5p reduced the effect of knockdown of MEG8 (*P* < 0.05) (Fig. 4a, b). Moreover, the depletion of MEG8 inhibited, and the miR-15a-5p and miR-15b-5p inhibitor promoted migration and invasion of A549 cells, in which the impact of MEG8 depletion was impaired by miR-15a-5p and miR-15b-5p inhibitor (Fig. 4c, d). CCK-8 assays revealed that the depletion of MEG8 reduced (*P* < 0.05), and the inhibitors of miR-15a/b-5p enhanced (*P* < 0.05) the cell viability in A549 cells, in which inhibitor of miR-15a-5p and miR-15b-5p attenuated the effect of MEG8 knockdown in the system (*P* < 0.05) (Fig. 5a). In addition, flow cytometry analysis showed that cell apoptosis was increased by the depletion of MEG8 (*P* < 0.01) in the cells, and miR-15a/b-5p inhibitors alleviated the influence of MEG8 depletion (*P* < 0.05) (Fig. 5b, c). These data suggest that lncRNA MEG8 induces NSCLC progression by targeting miR-15a/b-5p in vitro.

### MiR-15a-5p and miR-15b-5p inhibit NSCLC progression by targeting PSAT1 in vitro

**Fig. 6:**
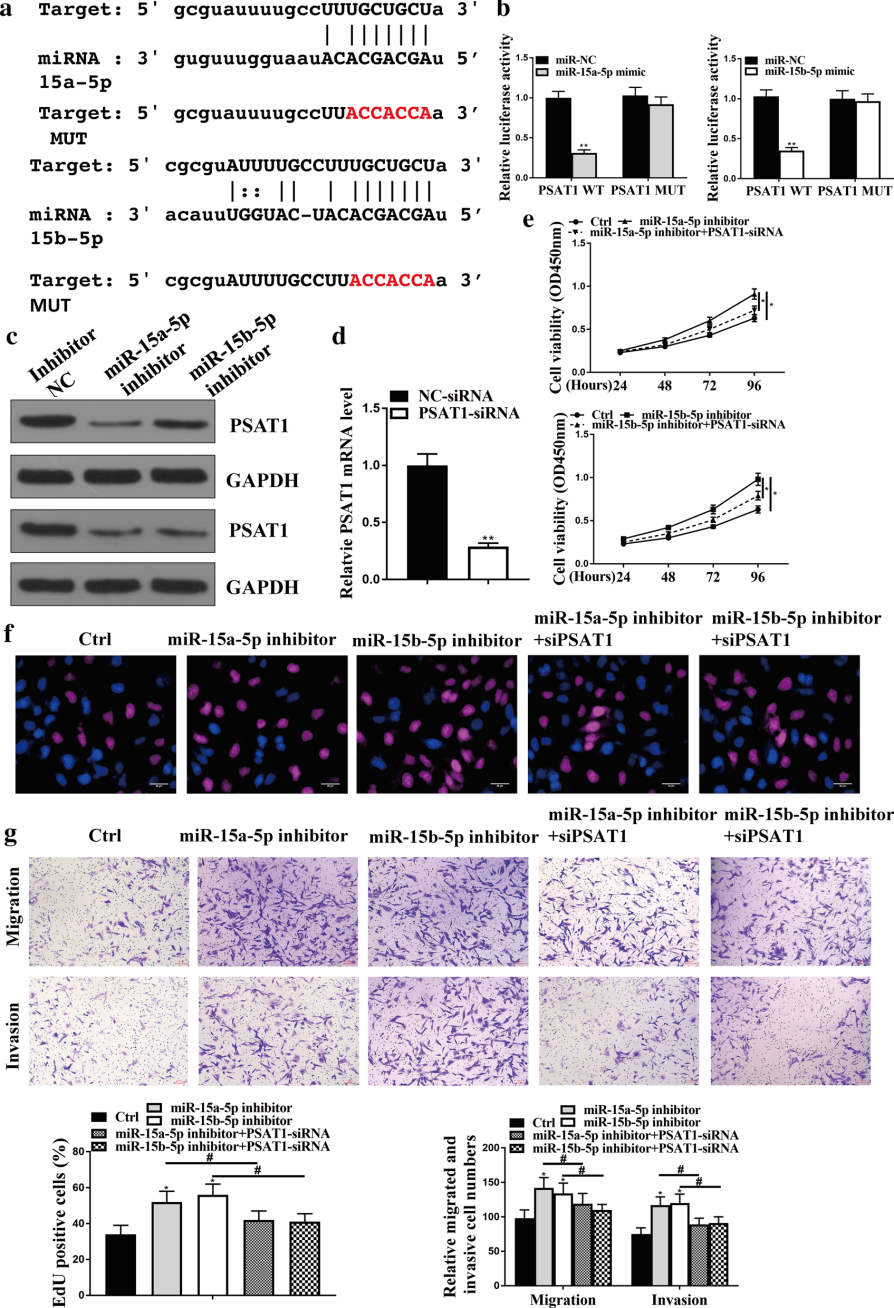
MiR-15a-5p and miR-15b-5p inhibit NSCLC progression by targeting PSAT1 in vitro. **a** The interaction of miR-15a/b-5p and PSAT1 3′ UTR was identified by bioinformatic analysis using Targetscan (http://www.targetscan.org/vert_72/). **b** The luciferase activities of wild type PSAT1 (PSAT1 WT) and PSAT1 with the miRNA-binding site mutant (PSAT1 MUT) were determined by luciferase reporter gene assays in the A549 cells treated with control mimic (miR-NC), miR-15a-5p mimic, or miR-15b-5p mimic, respectively. **c** The protein expression of PSAT1and GAPDH was tested by Western blot analysis in the A549 and H1299 cells treated with control inhibitor (inhibitor NC), miR-15a-5p inhibitor, or miR-15b-5p inhibitor, respectively. **d** The mRNA expression of PSAT1 was measured by qPCR in the A549 cells transfected with control siRNA (NC-siRNA) or PSAT1 siRNA (PAST1-siRNA). **e**–**g** The A549 cells were treated with control inhibitor (Ctrl), miR-15a/b-5p inhibitors, or co-treated with PSAT1 siRNA (PAST1-siRNA) and inhibitors of miR-15a/b-5p, respectively. **e** The cell viability was analyzed by CCK-8 assays in the cells. **f** The cell proliferation was tested by EdU assays in the cells. **g** The cell migration and invasion were examined by transwell assays in the cells. Data are presented as mean ± SD. Statistic significant differences were indicated: *P < 0.05, **P < 0.01, ^#^*P* < 0.05

Next, we tried to discover the target gene of miR-15a-5p and miR-15b-5p in NSCLC development in vitro. We identified the miR-15a-5p/miR-15b-5p-targeted site in the 3′-UTR of PSAT1 using Targetscan (http://www.targetscan.org/vert_72/) (Fig. 6a). And other targets of miR-15a-5p and miR-15b-5p were listed in Additional file 2. Notably, miR-15a-5p mimic along with miR-15b-5p mimic inhibited luciferase activities of the wild type PSAT1 but did not affect the PSAT1 with the miRNA-binding site mutant in A549 cells (*P* < 0.01) (Fig. 6b), which was shown in Fig. 6a. The protein expression of PSAT1 was down-regulated by the inhibitors of miR-15a/b-5p (Fig. 6c), suggesting that miR-15a/b-5p target to PSAT1. Similarly, the mRNA expression of PSAT1 was inhibited by miR-15a/b-5p inhibitors in the system (Additional file 1 Fig. 1B). Then, we transfected the PSAT1 siRNA and the control siRNA into A549 cells, and the efficiency was confirmed by qPCR assays (*P* < 0.01) (Fig. 6d). Moreover, CCK-8 assays (Fig. 6e) and EdU assays (Fig. 6f) showed that the depletion of PSAT1 impaired cell proliferation, which was enhanced by miR-15a/b-5p inhibitors in the cells (*P* < 0.05). Meanwhile, the inhibitors of miR-15a/b-5p promoted the migration and invasion of A549 cells, in which knockdown of PSAT1 could block these effects (*P* < 0.05) (Fig. 6g). Together these indicate that miR-15a-5p and miR-15b-5p inhibit NSCLC progression by targeting PSAT1 in vitro.

### MEG8 contributes to tumor growth of NSCLC via miR-15a/b-5p/PSAT1 axis in vivo

**Fig. 7:**
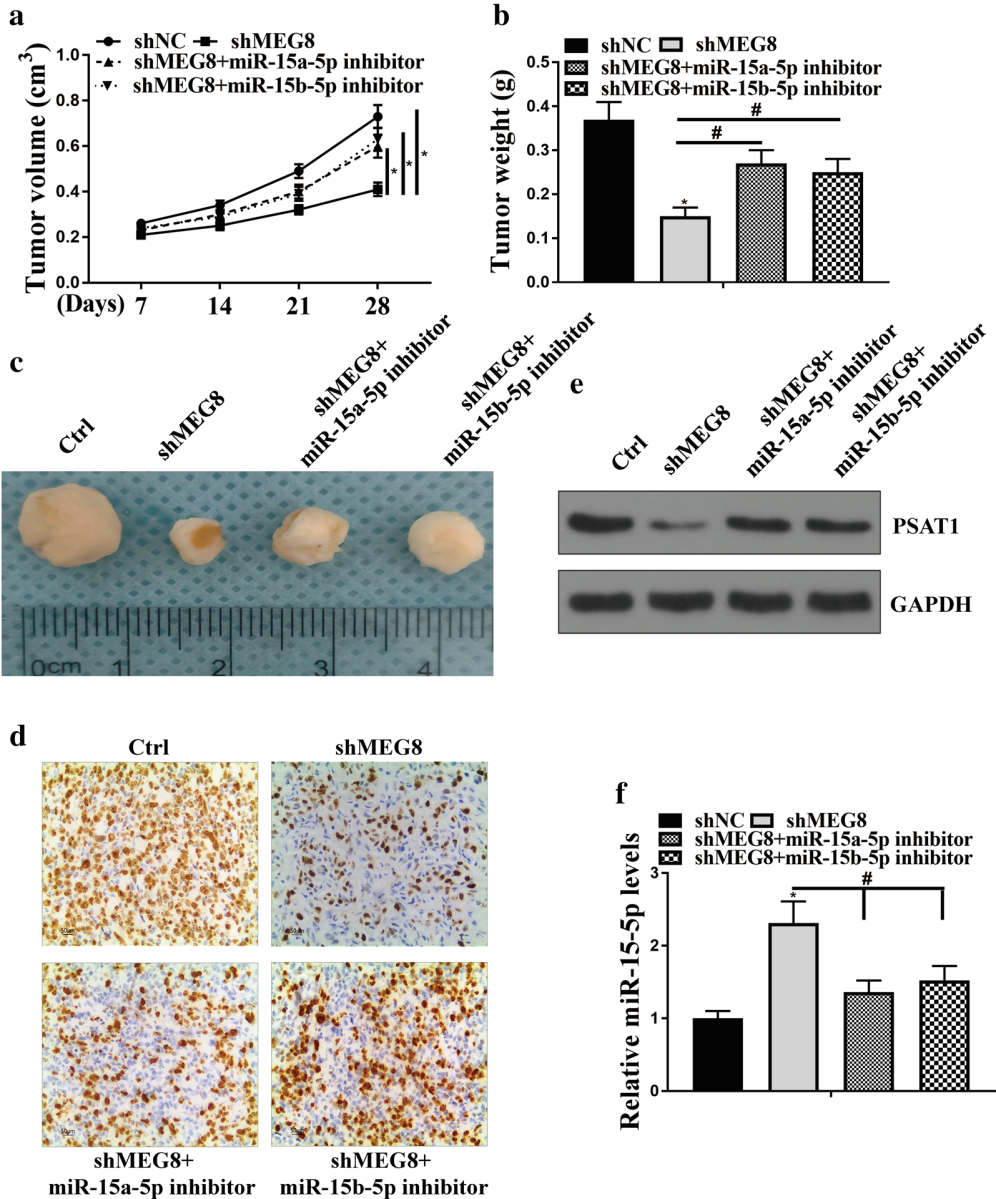
LncRNA MEG8 contributes to tumor growth of NSCLC via miR-15a-5p-miR-15b-5p/PSAT1 axis in vivo. **a**–**f** The effect of lncRNA MEG8-miR-15a-5p/miR-15b-5p axis on tumor growth of NSCLC cells in vivo was analyzed by nude mice tumorigenicity assay. The A549 cells were treated with control shRNA (Ctrl), MEG8 shRNA (shMEG8), or co-treated with MEG8 shRNA (shMEG8), and the inhibitors of miR-15a/b-5p and injected into the nude mice (n = 5). **a** The average tumor volume was calculated and shown. **b** The average tumor weight was calculated and shown. **c** Representative images of dissected tumors from nude mice were presented. **d** The expression levels of Ki-67 of the tumor tissues were measured by immunohistochemical staining. **e** The protein expression levels of PSAT1and GAPDH were examined by Western blot analysis in the tumor tissues. **f** The expression levels of miR-15-5p were tested by qPCR in the tumor tissues. Data are presented as mean ± SD. Statistic significant differences were indicated: *P < 0.05, ^#^*P* < 0.05

We further evaluated the effect of lncRNA MEG8-miR-15a-5p/miR-15b-5p axis on NSCLC development in vivo. Tumorigenicity analysis was performed in nude mice injected with A549 cells, which were treated with control shRNA, MEG8 shRNA, or co-treated with MEG8 shRNA and the inhibitors of miR-15a/b-5p. The depletion of MEG8 significantly reduced the tumor growth of A549 cells in vivo, in which the inhibitor of miR-15a-5p and miR-15b-5p could rescue the phenotype, as demonstrated by the tumor volume (*P* < 0.05) (Fig. 7a), tumor weight (*P* < 0.05) (Fig. 7b), tumor size (Fig. 7c) and the expression of Ki-67 in the lung tissues of mice (Fig. 7d). Besides, we validated that the attenuated expression of PSAT1 by knockdown of MEG8 was enhanced by the miR-15a/b-5p inhibitors in the lung tissue of mice (Fig. 7e). The increased expression level of miR-15-5p by knockdown of MEG8 was impaired by the miR-15a/b-5p inhibitors in the lung tissue of mice (*P* < 0.05) (Fig. 7f). Together these data suggest that lncRNA MEG8 contributes to tumor growth of NSCLC via the miR-15a-5p-miR-15b-5p/PSAT1 axis in vivo.

## Discussion

NSCLC is the predominant type of lung cancer with severe morbidity and high mortality [36]. As a crucial regulator in cancer development, lncRNAs are recognized to participate in the modulation of NSCLC. It has been reported that lncRNA UCA1 displays oncogenic roles in NSCLC by modulating miR-193a-3p [37]. LncRNA FEZF1-AS1 promotes epithelial-mesenchymal transition by regulating the WNT signaling and repressing E-cadherin in NSCLC [38]. LncRNA PCAT6 works as an oncogenic factor in NSCLC by regulating EZH2 and repressing LATS2 [39]. LncRNA AFAP1-AS1 foretells a worse NSCLC prognosis and modulates cell proliferation of NSCLC by epigenetically suppressing the expression of p21 [40]. LncRNA LINC-PINT restrains NSCLC development by targeting the miR-218-5p/PDCD4 signaling [41]. LncRNA MALAT1 contributes to the progression of NSCLC through regulating the miR-124/STAT3 signaling [42]. Besides, as a less studied lncRNA, lncRNA MEG8 is involved in the modulation of tumorigenesis. LncRNA MEG8 contributes to NSCLC progression by regulating the miR-107/CDK6 axis [43]. LncRNA MEG8 is associated with urological cancers [44]. In this study, we firstly identified that the expression levels of lncRNA MEG8 were elevated in NSCLC patients and NSCLC cell lines. The depletion of lncRNA MEG8 reduced NSCLC cell proliferation, migration, and invasion by targeting miR-15a/b-5p in vitro. LncRNA MEG8 contributed to tumor growth of NSCLC via miR-15a/b-5p/PSAT1 axis in vivo. These data present a novel function of lncRNA MEG8 in the NSCLC progression, providing valuable evidence for the fundamental role of lncRNAs in the development of NSCLC. The mechanism involving lncRNA MEG8, miR-15a/b-5p, and PSAT1 enriches the understanding of lncRNA MEG8, benefiting the future investigation of lncRNA MEG8 in other cancers.

As a primary component of ncRNA and the significant interplay factors with lncRNAs in the physiological and pathological processes, miRNAs are also involved in the modulation of NSCLC. MiR-760 represses the proliferation and metastasis of NSCLC by regulating the expression of ROS1 [45]. MiRNA-4735-3p-mediated FBXL3 inhibits cell proliferation and migration of NSCLC [46]. Circulating RNA 100,146 serves as an oncogene in NSCLC by interacting with miR-615-5p and miR-361-3p [47]. Circular microRNA-590-5p serves as a biopsy biomarker of NSCLC [48]. MiR-24 increases the invasion and migration of NSCLC by regulating ZNF367 [49]. MicroRNA-455 represses NSCLC by targeting ZEB1 [50]. MiR-451a decreases cell invasion and migration in NSCLC by modulating the expression of ATF2 [51]. Besides, miR-15b-5p and miR-15a-5p as the crucial modulator are involved in the regulation of cancer development by interacting with lncRNAs and targeted genes. LncRNA HOXA-AS2 promotes tumorigenesis by regulating the miR-15a-5p/HOXA3 signaling in papillary thyroid cancer [52]. SIRT1 represses the metastasis of colorectal cancer through transcriptional suppression of miR-15b-5p [53]. LncRNA MAGI2-AS3 regulates the expression of CCDC19 through targeting miR-15b-5p and overcomes the progression of bladder cancer [54]. The miR-15b-5p/PDK4 signaling controls the proliferation of osteosarcoma by the inflection of the Warburg effect [55]. MiR-15b-5p promotes tumorigenesis of prostate cancer by modulating the expression of RECK [56]. The down-regulation of microRNA-15a-5p represses cervical cancer progression through targeting TP53INP1 [57]. Moreover, previous studies revealed that the expression levels of miR-15a-5p and miR-15b-5p are decreased in NSCLC patients and is associated with the NSCLC diagnosis and prognosis [24, 58, 59]. We revealed that the expression levels of miR-15a-5p and miR-15b-5p were elevated in NSCLC patients and NSCLC cell lines. Our mechanical investigation further demonstrated that miR-15a/b-5p inhibited NSCLC progression by targeting PSAT1, in which miRNAs could be sponged by lncRNA MEG8. These data display an unreported role of miR-15a/b-5p in the development of NSCLC, identifying the new upstream lncRNA MEG8 and downstream target PSAT1 in the modulation of NSCLC.

It has been identified that PSAT1 is involved in the development of multiple cancers. Overexpression of PSAT1 enhances chemoresistance and promotes cell growth of colon cancer [60]. Regorafenib provokes mortal autophagy of glioblastoma by maintaining PSAT1 [61]. G9A-mediated PSAT1 controlled supports cell proliferation of colorectal cancer [62]. Nf1 suppression increases Kras-driven lung adenocarcinoma and affects PSAT1-related glutamate dependency [63]. PSAT1 serves as a biomarker of worse prognosis of nasopharyngeal carcinoma [64]. TAZ/YAP induces the expression of PSAT1 in breast cancer [65]. TAZ/YAP induces the expression of PSAT1 in breast cancer [66]. A particular defect of PSAT1 represses experimental metastasis, invasion, and migration in triple-negative breast cancer [67]. MiR-424 targets PSAT1 and possesses an inhibited function in colorectal cancer [68]. Besides, knockdown of PSAT1 improves the sensitivity of glutamine-limiting situations for NSCLC cells [69]. NRF2 regulates the expression of PSAT1 by ATF4 to promote nucleotide and glutathione production in NSCLC [70]. PSAT1 is a critical component of BRAF inhibitor resistance of pancreatic cancer, melanoma, and NSCLC [71]. Our data showed that PSAT1 contributed to the progression of NSCLC and is targeted by miR-15a-5p and miR-15b-5p, which could be sponged by lncRNA MEG8 in the system. These data provide new evidence that PSAT1 serves as a crucial factor in the development of NSCLC.

## Conclusions

**Fig. 8:**
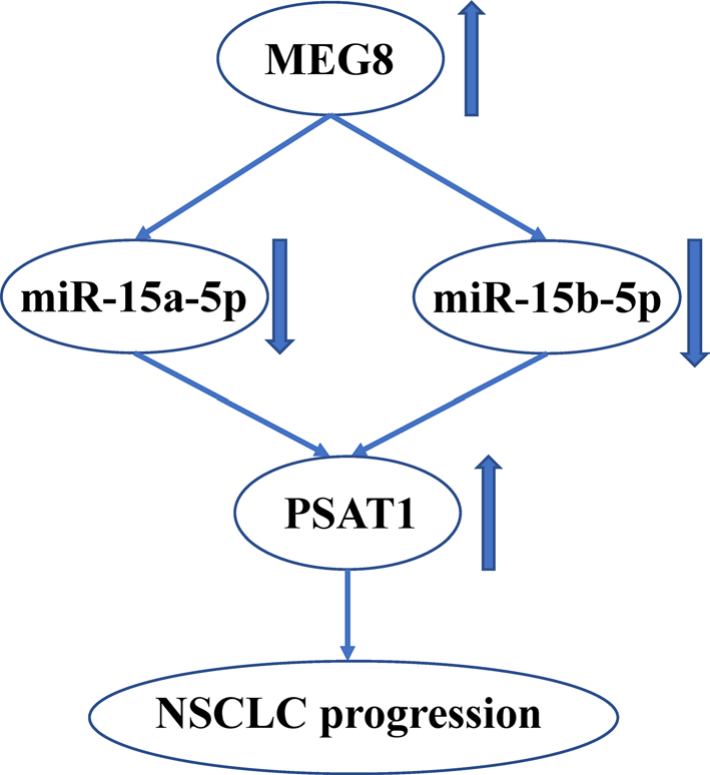
LncRNA MEG8 promotes NSCLC progression by modulating miR-15a-5p-miR-15b-5p/PSAT1 axis. The graphic model of this study was shown. Briefly, lncRNA MEG8 serves as a sponge of miR-15a-5p and miR-15b-5p in the NSCLC cells. MiR-15a-5p and miR-15b-5p is able to target PSAT1 in the NSCLC cells. LncRNA MEG8 up-regulates PSAT1 expression by sponging miR-15a-5p and miR-15b-5p. LncRNA MEG8 promotes NSCLC progression by modulating miR-15a-5p-miR-15b-5p/PSAT1 axis

In conclusion, we discovered that lncRNA MEG8 promoted NSCLC progression by modulating miR-15a-5p-miR-15b-5p/PSAT1 axis (Fig. 8). Our finding provides new insights into the mechanism by which lncRNA MEG8 contributes to the development of NSCLC, improving the understanding of lncRNA MEG8 and NSCLC. LncRNA MEG8, miR-15a-5p, miR-15b-5p, and PSAT1 may serve as potential targets for NSCLC therapy.

## Supplementary Information

**Additional file 1: Fig 1**. The effect of MEG8 on EMT markers and the effect of miR15a/b-5p on PSAT1. (A) The A549 and H1299 cells were infected with the lentiviral plasmids carrying MEG8 shRNA (shMEG8) or corresponding control shRNA (shNC). The expression of E-cadherin, N-cadherin, Vimentin, and GAPDH was measured by Western blot analysis in the cells and the results were quantified by ImageJ. (B) The mRNA expression of PSAT1 was tested by Western blot analysis in the A549 cells treated with control inhibitor (inhibitor NC), miR-15a-5p inhibitor, or miR-15b-5p inhibitor, respectively. Data are presented as mean ± SD. Statistic significant differences were indicated: **P < 0.01.

**Additional file 2**. Other targets of miR-15a-5p and miR-15b-5p were listed.

## Data Availability

The datasets generated and/or analyzed during the current study are not publicly available due research design, but are available from the corresponding author on reasonable request.

## Abbreviations

NSCLC: Non-small cell lung cancer
lncRNAs: Long non-coding RNAs
miRNAs: MicroRNAs
3′ UTR: 3′ Untranslated region
PSAT1: Phosphoserine aminotransferase 1
