# Supplementary figures and images for: LncRNA MEG8 promotes NSCLC progression by modulating the miR-15a-5p-miR-15b-5p/PSAT1 axis
Source: Cancer Cell Int. 2021 Feb 1;21:84. doi: 10.1186/s12935-021-01772-8 (PMC7852147; doi:10.1186/s12935-021-01772-8)

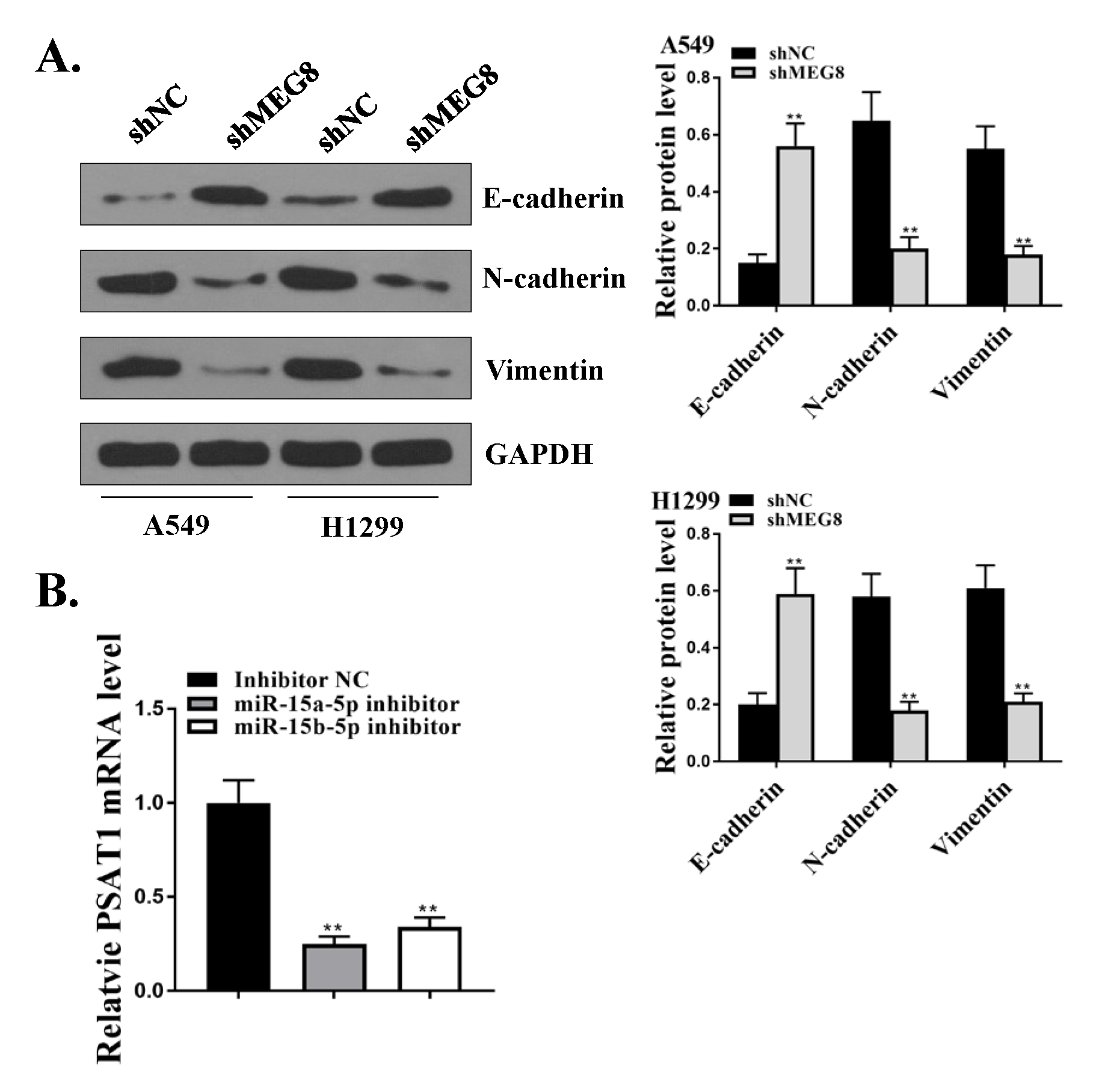

Supplement: Supplementary file 1 — Additional file 1: Fig 1. The effect of MEG8 on EMT markers and the effect of miR15a/b-5p on PSAT1. (A) The A549 and H1299 cells were infected with the lentiviral plasmids carrying MEG8 shRNA (shMEG8) or corresponding control shRNA (shNC). The expression of E-cadherin, N-cadherin, Vimentin, and GAPDH was measured by Western blot analysis in the cells and the results were quantified by ImageJ. (B) The mRNA expression of PSAT1 was tested by Western blot analysis in the A549 cells treated with control inhibitor (inhibitor NC), miR-15a-5p inhibitor, or miR-15b-5p inhibitor, respectively. Data are presented as mean ± SD. Statistic significant differences were indicated: **P < 0.01. [file 12935_2021_1772_MOESM1_ESM.tiff]
