# Supplementary material for: LncRNA MEG8 promotes NSCLC progression by modulating the miR-15a-5p-miR-15b-5p/PSAT1 axis
Source: Cancer Cell Int. 2021 Feb 1;21:84. doi: 10.1186/s12935-021-01772-8 (PMC7852147; doi:10.1186/s12935-021-01772-8)
Supplement: Supplementary file 2 — Additional file 2. Other targets of miR-15a-5p and miR-15b-5p were listed. [file 12935_2021_1772_MOESM2_ESM.docx]

**miR-15a-5p binding target**

MZF1-AS1 LINC00943 LINC00641 LINC00473 IQCH-AS1 MEG3 MEG8 HCG18 LINC00639 ZMIZ1-AS1 DLG1-AS1 XIST SNHG1 FGD5-AS1 NEAT1 SNHG16 MCM3AP-AS1 LINC00662 SNHG25 LINC02035 LINC01184 C1RL-AS1 PAST1

**miR-15b-5p binding target**

XIST SNHG1 FGD5-AS1 NEAT1 SNHG16 LINC00662 LINC01184 LINC02035 LINC00649 SNHG12 NUTM2A-AS1 C1RL-AS1 MIR29B2CHG HCG18 TTN-AS1 LINC00852 HCG17 LINC00852 SLFNL1-AS1 LINC00473 LINC00641 LIPE-AS1 TEX41 LINC00511 IQCH-AS1 PAST1
